# A CONVERGENT PARALLEL MIXED METHODS ANALYSIS OF SUCCESSFUL AGING IN CHINESE AMERICANS: DOES ACCULTURATION MATTER?

**DOI:** 10.1093/geroni/igad104.1221

**Published:** 2023-12-21

**Authors:** Mushira Khan, Ezer Kang, Chien-Ching Li

**Affiliations:** Mather Institute, Evanston, Illinois, United States; Howard University, Washington, District of Columbia, United States; Rush University, Chicago, Illinois, United States

## Abstract

Chinese Americans comprise the largest Asian origin group aged 65 years and older (26%), yet little is known about how aging, particularly in the context of acculturation, is experienced within this group. Further, although immigration scholarship underscores a robust, positive relationship between successful aging and acculturation, how major lifecourse events shape perceptions of aging well in Chinese Americans is not well-understood. Using an intersectional lifecourse perspective, this convergent parallel mixed methods study presents integrated findings from a cross-sectional survey (N=98; 58.2% female; mean age=64.4 years) and qualitative interviews (N=32; 53% female; mean age=59.4 years) with community-dwelling Chinese Americans 50+, about 80% of whom were foreign-born. Survey measures included the Short Acculturation Scale, the Successful Aging Scale, self-reported health, and demographic variables. Quantitative data were analyzed using multiple regression analysis. No significant association was found between acculturation and successful aging, even after controlling for length of stay and sociodemographic variables. Thematic analysis of qualitative data revealed feelings of ambivalence and alienation. Several participants shared that despite having lived in the US for decades, they felt like they “no longer belonged”, feared for their safety, and felt a “sense of betrayal” after the recent increase in Anti-Asian hate crimes. Many were planning to return to their home country, where they believed it would be easier to age successfully. These findings suggest that important turning points in the lifecourse trajectory, such as experiences of racism, can negatively influence perceptions of aging in Chinese Americans, regardless of degree of acculturation and length of stay.

